# Elevated serum growth differentiation factor 15 levels are associated with thyroid nodules in type 2 diabetes aged over 60 years

**DOI:** 10.18632/oncotarget.17328

**Published:** 2017-04-21

**Authors:** Hongmei Zhang, Weiwei Zhang, Xiaofang Tu, Yixin Niu, Xiaoyong Li, Li Qin, Zhen Yang, Qing Su

**Affiliations:** ^1^ Department of Endocrinology, Xinhua Hospital Affiliated to Shanghai Jiaotong University School of Medicine, Shanghai 200092, China

**Keywords:** thyroid nodule, growth differentiation factor 15, type 2 diabetes

## Abstract

In order to investigate whether serum growth differentiation factor 15 is associated with thyroid nodules in type 2 diabetes. We recruited 723 type 2 diabetic patients aged over 30 years who attended the clinic of Endocrinology of Xinhua Hospital from January 2013 to January 2015. Thyroid nodule was diagnosed by thyroid ultrasonographic examination. Serum growth differentiation factor 15, thyroid function, thyroid autoantibodies, thyroglobulin and other biochemical indicators were measured and compared between thyroid nodule positive and negative groups. We found that overall, serum growth differentiation factor 15 levels were significantly higher in subjects with thyroid nodules compared with nodule negative subjects (181.76±98.49 pg/ml vs. 162.32±83.63 pg/ml, p<0.05), and this was influenced by age. In the patients over 60 years, this difference became more significant (211.23±103.66 pg/ml vs. 177.38±85.51 pg/ml, p<0.01), but in patients under 60 years, there was no difference between the two groups. Multivariate logistic regression analysis showed that serum growth differentiation factor 15 levels were independently associated with thyroid nodule in diabetic patients over 60 years (P <0.001). After multiple adjustments, the odds ratios were substantially higher for thyroid nodule (odds ratio 2.63, 95% confidence interval 1.30-5.13, p<0.01) in the highest growth differentiation factor 15 quartile compared to those in the lowest quartile in patients over 60 years. In conclusion, serum growth differentiation factor 15 is increased significantly in subjects with thyroid nodules in type 2 diabetic patients aged over 60 years.

## INTRODUCTION

Growth differentiation factor 15 (GDF-15) is structurally similar to the human transforming growth factor β (TGF-β), also being called nonsteroidal anti-inflammatory drug (NSAID)-activated gene-1 (NAG-1), macrophage inhibitory cytokine 1 (MIC-1), prostate differentiation factor (PDF), placental bone morphogenetic protein (PLAB), and placental TGF-β (PTGF-β) [1]. In the normal physiological states, the expression level of GDF-15 is very low in the majority cell types and tissues except for the macrophages and placenta [2]. In conditions of acute injury, inflammation, and cancer, GDF-15 will be expressed at a dramatically high level [3–5]. GDF-15 is overexpressed in numerous types of tumors such as colon cancer, prostate cancer, pancreatic cancer, breast cancer, and thyroid carcinomas [6, 7]. Measuring GDF-15 levels can be used in the diagnosis and management of the diseases. Elevated expression levels inside the primary tumor are related to increased serum levels of GDF-15, suggesting a constant release of GDF-15 from the primary tumor into the blood flow [8]. Actually, serum GDF-15 levels are often increased in patients suffering from different types of cancer, including ovarian cancer, pancreatic cancer, and prostate cancer. Therefore, GDF-15 seems to be involved in regulating tumor cell growth.

Thyroid nodule is one of the common benign thyroid disorders [9]. With the widely used technology of modern ultrasound the prevalence of thyroid nodules has risen up to 76 % [10], but the molecular mechanisms of thyroid nodules are still not clear. The natural occurring thyroid growth heterogeneity could provide a reasonable explanation for the early stages of nodule formation. If there is a high inherent growth potential in a thyrocyte or if the thyrocyte is affected by some protooncogene overexpression or some growth factor or it is hit by some oncogene or other molecular events, the cell will proliferate to form a tumor [11].

Diabetes mellitus (DM) and thyroid disease are the two most common diseases in the endocrine system [12]. Currently, a meta-analysis of 10920 diabetic patients has shown that the frequency of thyroid disease was 11% [13]. Some researchers have found that patients with type 2 DM had increased thyroid volumes and elevated prevalence of thyroid nodule [14], but research about the connection between type 2 DM and thyroid nodule are still sparse. Some studies revealed that circulating levels of GDF-15 in patients with recognized type 2 diabetes were much higher [15, 16], and GDF-15 is related to increased cancer risk in type 2 diabetes [17]. Since GDF-15 has potential cell growth promoting activity and its circulating concentrations in type 2 diabetes are elevated, it is logical to regard GDF-15 as a promising candidate for risk assessment for thyroid nodule in type 2 diabetic patients. To better evaluate the possible role of GDF-15 in thyroid nodule development in type 2 diabetes, we examined the relationship between serum GDF-15 levels and thyroid nodule in type 2 diabetic patients.

## RESULTS

### Baseline characteristics

**Figure 1:**
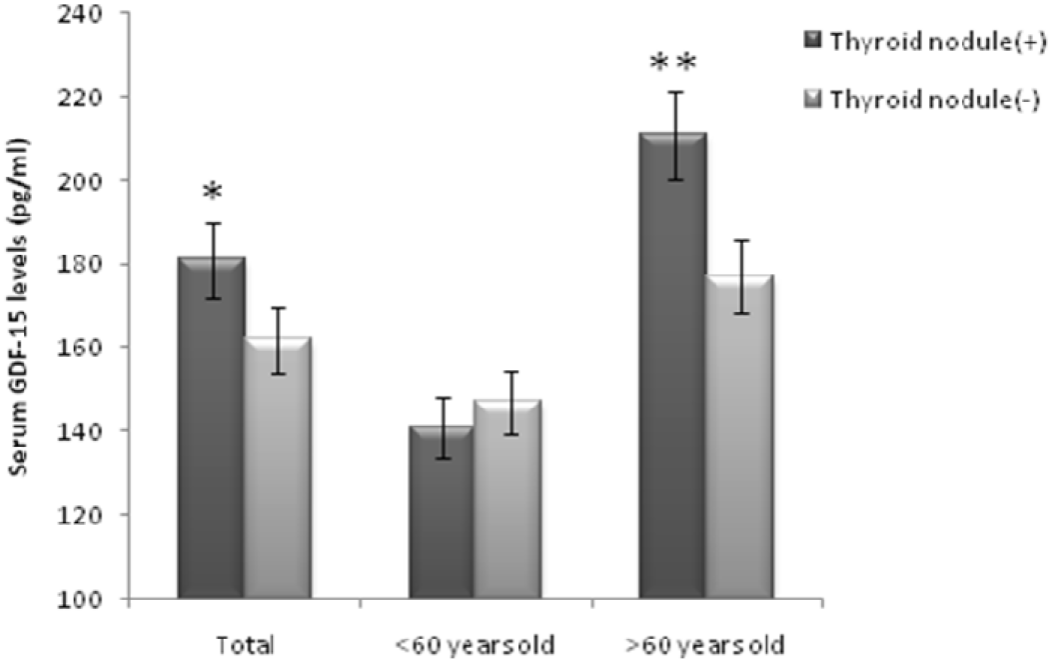
Serum GDF-15 levels in thyroid nodule positive and thyroid nodule negative groups Data are shown as means ± SD. *p<0.05, **p<0.01.

Of the 723 studied participants, 402 subjects were thyroid nodule positive (226 male). As shown in Figure 1, the serum GDF-15 levels were significantly higher in subjects with thyroid nodules compared with nodule negative subjects (181.76±98.49 pg/ml vs. 162.32±83.63 pg/ml, p<0.05). After being divided by age, this difference became more significant (211.23±103.66 pg/ml vs. 177.38±85.51 pg/ml, p<0.01), but in patients under 60 years, there was no difference between the two groups. The clinical characteristics of participants were shown in Table 1. When compared with thyroid nodule negative subjects, the thyroid nodule positive ones had significantly older age (p<0.001), lower FT3 and FT4 levels (p<0.05), and increased thyroid volumes (p<0.05).

**Table 1:** Clinical and laboratory characteristics of study subjects

Characteristics	Thyroid nodule (+)	Thyroid nodule (-)	P value
N	402	321	
Age (year)	63.59±11.78	58.08±13.69	<0.001
Sex (Male/Female)	226/176	206/115	0.053
BMI (kg/m^2^)	24.84±3.86	25.03±3.99	0.538
SBP (mm Hg)	133.01±15.85	131.78±15.54	0.317
DBP (mm Hg)	76.93±9.72	77.79±10.15	0.264
TT3 (nmol/l)	1.45±0.40	1.99±0.77	0.436
TT4 (nmol/l)	115.21±21.07	117.98±28.26	0.174
FT3 (pmol/l)	4.41±0.74	4.58±1.11	<0.05
FT4 (pmol/l)	17.49±3.52	18.39±5.12	<0.05
TSH (uIU/ml)	1.75 (0.94-2.57)	1.82 (0.77-2.71)	0.371
TPOAb (IU/ml)	35.90 (28.00-47.60)	36.60 (28.78-61.53)	0.223
TRAb (IU/ml)	0.30 (0.30-0.61)	0.32 (0.30-1.09)	0.870
TGAb (U/ml)	18.95 (14.60-32.40)	21.90 (17.23-47.7)	0.535
Tg (ng/ml)	8.24 (4.17-13.28)	7.08 (4.35-20.73)	0.088
Thyroid Volume (mL)	8.39±4.32	7.42±3.25	<0.05
Insilin (pmol/l)	98.35±18.36	81.48±15.24	0.133
TG (mmol/l)	1.94±0.31	2.34±0.56	0.150
CHO (mmol/l)	4.74±1.16	4.72±1.06	0.806
LDL (mmol/l)	2.85±0.79	2.83±0.76	0.790
HDL (mmol/l)	1.36±0.48	1.60±0.44	0.436
HbA1C (%)	9.68±2.33	10.10±3.58	0.219
FBS (mmol/l)	8.75±3.33	8.40±3.02	0.267

Data are means ± SD, median (interquartile range) or number (percent).

Correlation analysis showed a remarkable correlation between GDF-15 and age (r=0.351, p<0.001); GDF-15 and log_10_Tg (r=0.138, p<0.01); GDF-15 and CHO (r=-0.158, p<0.001); GDF-15 and LDL (r=-0.161, p<0.001) (see Table 2). A multiple stepwise regression analysis was used to evaluate the independent variables that may have an effect on GDF-15 serum levels. The main determinants of GDF-15 are age (β=0.375, p<0.001); Tg (β=0.155, p<0.01) (see Table 3).

**Table 2:** Crude and partial correlation between serum GDF-15 and clinical parameters in the study subjects

Variable	Crude r	Partial r^†^
Age (year)	0.351**	-
BMI (kg/m^2^)	0.014	0.046
SBP (mmHg)	0.034	0.029
DBP (mmHg)	0.026	0.022
TT3 (nmol/l)	-0.071	-0.029
TT4 (nmol/l)	0.042	-0.083
FT3 (pmol/l)	-0.196**	-0.095
FT4 (pmol/l)	-0.049	-0.014
log_10_TSH (uIU/ml)	0.037	0.024
log_10_TPOAb (IU/ml)	-0.006	-0.002
log_10_TRAb (IU/ml)	-0.069	-0.048
log_10_TGAb (U/ml)	0.009	-0.033
log_10_Tg (ng/ml)	0.177**	0.138*
INS (pmol/l)	0.088	0.047
Triglycerides (mmol/l)	0.024	0.082
CHO (mmol/l)	-0.149*	-0.158**
LDL (mmol/l)	-0.123*	-0.161**
HDL (mmol/l)	-0.028	-0.020

*p < 0.01, **p < 0.001, †adjusted for age, gender.

**Table 3:** Multiple stepwise regression analysis showing variables independently associated with the serum level of GDF-15

Independent variables	Standardized β	t	P value
Age	0.375	7.285	<0.001
log_10_Tg	0.155	3.043	<0.01

The analysis also included gender, FT3, CHO, LDL which were all excluded in the final model.

### Association between GDF-15 and thyroid nodule

Since GDF-15 is correlated with age, and multiple logistic regression analysis indicated that age was independently associated with thyroid nodule (P<0.001) (Table 4). We further divided the subjects into two groups according to age at 60 years old, and we found that GDF-15 was independently associated with thyroid nodule in patients over 60 years (P<0.01) (Table 4). In patients under 60 years old, GDF-15 was not related to thyroid nodule.

**Table 4:** Multiple logistic regression analysis showing variables independently associated with thyroid nodule

	Independent variables	B	SE	Wald	Exp(B) 95% CI	P value
Total	Age	0.30	0.007	16.499	1.031 (1.00-1.05)	<0.001
>60 years	GDF-15	0.05	0.002	7.215	1.050 (1.01-1.07)	<0.01

The analysis also included age gender, BMI, FT3, FT4, thyroglobulin, insulin, TG, and thyroid volume which were all excluded in the final model.

Table 5 displays the ORs for thyroid nodule according to GDF-15 quartiles (≤106.24; 106.25–149.79; 149.80–214.08; and ≥214.09 pg/ml, respectively). As assumed, we found increased ORs for thyroid nodule from the 1st to the 4th GDF-15 quartiles (P< 0.01 for trend). In the highest GDF-15 quartile, the adjusted ORs for thyroid nodule was 2.63 (95% CI, 1.30 to 5.13) after adjusting for gender, FT3, FT4, and thyroid volume.

**Table 5:** Adjusted ORs and 95% CIs for thyroid nodule according to GDF-15 quartiles in subjects

	ORs (95% CI)				P value for trend
	Q1 (pg/ml)≤106.24	Q2 (pg/ml)106.25–149.79	Q3 (pg/ml)149.80–214.08	Q4 (pg/ml)≥214.09	
>60 years					
Model 1	1	1.39 (0.66-2.93)	1.45 (0.79-2.64)	2.45(1.33-5.19)	<0.01
Model 2	1	1.37 (0.63-2.90)	1.41 (0.76-2.61)	2.63 (1.30-5.13)	<0.01
≦60 years					
Model 1	1	0.9(0.41-2.21)	1.06(0.48-2.35)	1.45(0.70-2.99)	>0.05
Model 2	1	0.77 (0.32-1.86)	0.85(0.37-1.96)	1.28 (0.61-2.69)	>0.05

Q, Quartile.

Model 1 unadjusted.

Model 2 adjusted for gender, FT3, FT4, and thyroid volume.

## DISCUSSION

The major new finding of the present study is that serum GDF-15 level is positively associated with thyroid nodule in type 2 diabetic patients aged over 60 years. These results are consistent with the hypothesis that serum GDF-15 is a determinant of thyroid nodule in type 2 diabetic patients aged over 60 years. Our findings suggest that serum GDF-15 may be involved in the higher thyroid nodule risk observed in elderly type 2 diabetic subjects.

GDF-15, also known as macrophage inhibitory cytokine 1, was described as a distinct member of TGF-β superfamily [1]. GDF-15 expression and secretion are both increased in some malignant tissues and cancer cell lines when compared with their normal tissues or cells [18, 19]. In addition, the serum GDF-15 levels in human cancer patients are high, with declining patient survival. Measurement of serum GDF-15 level has been proposed as an indicator for cancer progression and risk assessment [20]. The intuitive response is to consider the highly expressed GDF-15 protein as a driving factor in tumor growth. GDF-15 can stimulate the activation of Akt and the extracellular signal-related kinase (ERK) pathway [21]. Except for its direct bioactivity in cancer cells, GDF-15 has frequently been reported to modulate the tumoral microenvironment [22] and regulate the immunization. Pro-angiogenesis could be another way for GDF-15 to promote tumor growth. Some research indicated that GDF-15 is related to malignant thyroid follicular neoplasia [23], but so far there is no evidence to imply the association between GDF-15 and begin thyroid nodule. Our results supported that GDF-15 may be involved in the formation of thyroid nodules in type 2 diabetes. And thyroglobulin is independently associated with GDF-15. As far as we know, this is the first study to link GDF-15 and thyroid nodule. Large-scale investigations will be needed to testify this connection in the future.

Thyroid nodule is a common disease in clinical practice. The accurate mechanism of thyroid cell proliferation remains unclear. It was reported that impaired glucose metabolism is a risk factor for enlarged thyroid and increased nodule prevalence [14]. Other studies have also revealed that type 2 DM has an effect on the formation of thyroid nodules [24, 25]. Patients with type 2 DM had larger thyroid and elevated risk of thyroid nodule formation [14]. The reason for having higher thyroid nodule prevalence in diabetic patients is uncertain. Some studies reported that circulating concentrations GDF-15 in patients with type 2 diabetes were much higher [15, 16], and GDF-15 is related to cell hyperplasia in type 2 diabetes [17]. It is logical to consider GDF-15 as a risk factor for thyroid cell proliferation in diabetes. Our findings confirmed this, we found serum GDF-15 levels were independently associated with thyroid nodule in diabetic patients over 60 years.

Hypothyroidism is a well-known stimulator of thyroid growth [26]. Another mechanism involved in thyroid cell proliferation is thyroid autoimmunity [27]. The Hashimoto thyroiditis includes proliferating nodules formation, cytological alterations, and nuclear modification similar to papillary thyroid carcinoma [28]. Although we found decreased FT3 and FT4 levels in the subjects with thyroid nodule, but after adjusting age and other confounders, FT3 and FT4 levels were not related to thyroid nodules which means the lower levels of FT3 and FT4 may be caused by old age, which is consistent with previous studies [29]. In the present study, maybe because of the sample size, we didn't find hypothyroidism and thyroid autoimmunity were related to thyroid nodule. And we didn't find GDF-15 was associated with hypothyroidism and thyroid autoimmunity either.

Our study has some limitations. Firstly, there was not enough information about the morphological characteristics of thyroid nodules such as nodule diameters and uni-/multinodularity. Secondly, the cytological and/or histopathological outcome of each thyroid nodule has not been assessed. Including these assessments may improve the strength of future studies. Thirdly, because the subjects were from the clinic of our hospital, a single center in China, the study population may not represent the general population.

## SUBJECTS AND METHODS

### Subjects

The participants were from the clinic of Endocrinology of Xinhua Hospital Affiliated to Shanghai Jiaotong University School of Medicine between January 2013 and January 2015. We recruited 723 patients (432 men and 291 women) in total. All of the 723 patients were type 2 diabetic and aged over 30 years. Diabetes was diagnosed according to WHO 1999 diagnostic criteria. All participants have a diabetes history of more than 3 years and were under hypoglycemic treatments. Patients with severe cardiovascular disease, renal insufficiency, liver dysfunction were excluded. Informed consent was obtained from all of the participants. The research protocol was approved by the Ethics Committee of Xinhua Hospital Affiliated to Shanghai Jiaotong University School of Medicine.

### Thyroid parameters measurements and clinical data collection

After overnight fasting, peripheral venous blood samples were collected. Thyroid function, thyroid antibodies, thyroglobulin, and insulin were assayed by an automated analyzer (ADVIA Centaur XP, Siemens, Berlin, German). HbA1c was determined by high-performance liquid chromatography (BIO-RAD VARIANT II, California, USA). Blood lipids were measured with an autoanalyzer (Hitachi 7600, Tokyo, Japan). Body mass index (BMI) was calculated as weight in kilograms divided by the square of height in meters. Blood pressure was measured three times from the right arm of patients after 30 min resting in a sitting position; then mean values were calculated.

### Measurement of GDF-15

Serum samples were isolated and stored at -80°C until analysis. The serum GDF-15 levels were detected by ELISA with Duoset kit (DY957; R&D Systems, Minneapolis, MN) in duplicate according to the recommendations of the manufacturer. The ELISA system possesses an inter-assay coefficient of variation of 4-10% and intra-assay coefficient of variation of 3-9%, respectively.

### Thyroid nodule measurement

Thyroid ultrasonographic examination was conducted by two experienced ultrasonographists using high-resolution B-mode tomographic ultrasound system (Esaote Biomedica SpA) with 10-MHz transducers. Thyroid nodules are defined as discrete lesions within the thyroid gland that are radiologically different from the surrounding thyroid tissue [30]. Each nodule was measured in three dimensions. The smallest diameter measured is 2-3mm. We included solid lesions and mixed cystic-solid lesions, but cystic lesions were excluded. Thyroid volume was calculated using the ellipsoid formula: volume (mL)=depth (cm)×width (cm)×length (cm)× π/6 [14].

### Statistical analysis

Normally distributed data were shown as means ± SD, non-normally distributed data were expressed as median (interquartile range) and log-transformed to approximate normality prior to analysis. Unpaired Student's t-test was used to test differences between groups. Correlation coefficients between GDF-15 and other parameters were calculated by Pearson correlation analysis. Multiple stepwise regression analysis was used to determine the relationship between serum GDF-15 and other parameters. Multiple logistic regression models were used to calculate the odds ratios (ORs) for thyroid nodule. Variables entered in the model were chosen according to the clinical and statistical significance. Any variable having a significant univariate test at 0.20 was selected as a candidate for the multivariate analysis. In the end, variables included in the model were age, gender, BMI, FF3, FT4, thyroglobulin, insulin, TG, and thyroid volume. Covariates were removed from the model if they were non-significant and not a confounder. We divided the subjects into two groups by age at 60 years old and analyzed the associations of GDF-15 and thyroid nodule in different age groups. All statistical analysis was conducted using SPSS Statistical Package (version 13.0; SPSS Inc., Chicago, IL). P values < 0.05 were considered statistically significant.

## CONCLUSION

In conclusion, our study suggests that serum GDF-15 is a marker for the development of thyroid nodule in patients with type 2 diabetes over 60 years old. Measurement of serum GDF-15 could be useful for thyroid nodule risk stratification in the diabetic population.

## Abbreviations

GDF-15: Growth differentiation factor 15
BMI: body mass Index
SBP: systolic blood pressure
DBP: diastolic blood pressure
TT3: total triiodothyronine
TT4: total thyroxine
FT3: free triiodothyronine
FT4: free thyroxine
TSH: thyrotropin
TPOAb: antithyroid peroxidase antibodies
TGAb: anti-thyroglobulin antibody
TRAb: thyrotropin receptor antibodies
Tg: thyroglobulin
